# Negative Regulation of PTEN by MicroRNA-221 and Its Association with Drug Resistance and Cellular Senescence in Lung Cancer Cells

**DOI:** 10.1155/2018/7908950

**Published:** 2018-01-30

**Authors:** Ning Wang, Chen Zhu, Ye Xu, Wenliang Qian, Min Zheng

**Affiliations:** Department of Thoracic Surgery, Tongren Hospital, Shanghai Jiao Tong University School of Medicine, Shanghai, China

## Abstract

**Objective:**

Chemotherapy is the routine method for treating many cancers, but long-term treatment may result in developing resistance to the drugs. The aim of this study was to identify whether noncoding RNAs play a role in drug resistance and how they affect drug resistance.

**Materials and Methods:**

The expression levels of miR-221 in different lung cancer cell lines H226, H1299, and A549 were measured. H1299 and A549 cell lines were transfected to overexpress and downexpress miR-221, and cell viability and cell senescence were determined. The PTEN/Akt pathway was then examined by real-time polymerase chain reaction and Western blot analysis.

**Results:**

MiR-221 together with proteins MDR1 and ABCG2 was upregulated in Cisplatin-resistant A549 lung cancer cells. Anti-miR-221 inhibits proliferation and induces senescence in lung cancer cells. PTEN/Akt pathway axis was identified as a target of drug resistance induced by miR-221.

**Conclusion:**

Our results revealed that miR-221 is an important regulator for chemotherapy sensitivity and showed miR-221 as a potential target for drug sensitization.

## 1. Introduction

Although great strides have advanced the treatment of many cancers in recent decades, drug resistance creates a major obstacle for optimal treatment and often causes relapse. Therefore, detailed exploration of the drug resistance mechanisms will be of much benefit for improving the results of chemotherapy. Recent studies show that aberrant microRNA expression is closely related to drug resistance of cancer patients [1].

Of all cancers, lung cancer is the most common worldwide, and every year more cases are reported [2]. In the majority of these cases, activation of the proto-oncogene and inactivation of the tumor suppressor gene affect the development and progression of epithelial cancers. However, a recent study revealed that microRNAs (miRNAs) may be able to regulate gene expression by specifically targeting mRNA 3′ untranslated region (3′UTR) with resulting inhibition of mRNA translation and mRNA degradation [3]. Since an individual miRNA may regulate many different mRNAs, many thousands of human miRNAs are suspected of modulating more than one-third of the mRNA species encoded in the whole human genome. They also play an important role in tumorigenesis [4]. Moreover, the involvement of miRNAs in many physiological processes such as cell growth, proliferation, apoptosis, differentiation, and receptor-driven pathways [5] could affect the effectiveness of chemotherapy [6]. However, how patients respond to chemotherapy varies widely. Recent studies have shown that miRNAs are key players in the development of chemotherapy resistance [7–9]. miRNAs are differentially expressed in chemosensitive and chemoresistant cells.

Among oncogenic microRNAs, miR-221 and miR-222 (miR-221/222) carry the same sequence. This sequence is evolutionarily conserved and frequently binds short regions at its targeting gene 5′ ends. Many studies indicate that these two miRNAs often target several high expression genes in epithelial cancers such as glioma, prostate carcinoma, hepatocellular cancer, and breast cancer [10–13]. Cisplatin is one of the major chemotherapeutic regimens in lung cancer treatment. Despite initial clinical response, patients may eventually develop resistance to this chemotherapy. So far, the resistance mechanism for Cisplatin in lung cancer is not clear. Our research aimed to investigate the role of miR-221 in lung cancer cells, especially its role and mechanism in drug resistance.

In this study, we identified the PTEN/Akt pathway axis as a target of miR-221-induced cellular senescence. Our results revealed the role of miR-221 in regulation of chemosensitivity and showed miR-221 as a potential target for drug sensitization.

## 2. Materials and Methods

### 2.1. Cell Culture and Transfection

Human lung cancer cell lines H1299, H226, and A549 were maintained in Dulbecco's modified Eagle's medium (DMEM) (Gibco, USA) supplemented with 10% fetal bovine serum (Gibco), 2 mM glutamine (Sigma), 100 units of penicillin/ml (Sigma), and 100 *μ*g of streptomycin/ml (Sigma). The cells were incubated at 37°C with 5% CO2. 2′-O-methyl- (OMe-) oligonucleotides were chemically synthesized and purified by high-performance liquid chromatography by GenePharma Co. Ltd. (Shanghai, China). All the bases were 2′-OMe modified entirely and had the following sequences: 2′-OMe-anti-miR-221(As-miR-221), 5′-AGCUACAUUGUCUGCUGGGUUUC-3′; 2′-OMe-oligonucleotides (200 pmol) were transfected using Lipofectamine 2000 (Invitrogen). Scrambled oligonucleotides (Scr) were also transected as a control and had the following sequence: 5′-UCUACUCUUUCUAGGAGGUUGUGA-3′. All the functional detections were done three days after transfection. Cellular senescence was determined by Senescence-associated beta galactosidase (SA-*β*-gal) assay as described [12].

### 2.2. Induction of Cisplatin- (CDDP-) Resistant Lung Cancer Cell Lines

To induce CDDP-resistant A549 lung cancer cell lines, a progressive concentration of CDDP was used. Then the A549 cells in logarithmic growth were treated briefly with 0.25 *μ*g/mL of CDDP. After 48 hours, CDDP was removed and cells were cultured without CDDP until they recovered. The same treatment was repeated until the cells were resistant to the current concentration, at which time the CDDP concentration was gradually increased to 0.5, 0.75, and finally 1.0 *μ*g/mL. The induced cells lived in 6 *μ*mol/L of CDDP for about 2 months with normal activity and continued normal activity after having CDDP withdrawn. They exhibited characteristics of CDDP resistance under the conditions of 1 *μ*g/ml CDDP drugs in the culture medium, so the cells were confirmed to be CDDP resistant and named A549/CDDP.

### 2.3. Cell Senescence Assay

Cell senescence was determined using a SA-*β*-gal assay kit (Genmed, USA), following the instructions described by the company.

### 2.4. Plate Colony Formation

To obtain plate colony formation assay, cells were seeded in a six-well plate (1 × 10^3^ cells/well), incubated at 37°C for 14 days, and then washed twice in PBS. They were stained with 0.5% crystal violet solution to create images for observation.

### 2.5. Cell Viability Assay

Cells were seeded into 96-well plates at 4,000 cells/well. For H1299-miR-221 and A549-anti-miR-221 cells, after transfection as described previously, 20 *μ*l of CCK8 was added to each well each day for 5 consecutive days after treatment and then incubated for 4 hours. The supernatant was then discarded and the precipitate dissolved by adding 200 *μ*l of DMSO to each well. Optical density (OD) was measured at the wavelength of 570 nm. The data are presented as the mean ± SD, derived from triplicate samples of at least three independent experiments.

### 2.6. Western Blots

Using a 10% SDS-polyacrylamide gel, equal amounts of protein per lane were separated and moved to PVDF membrane. The membrane was blocked in 5% skim milk for 1 hour and then incubated with a specific antibody for 2 hours. The antibodies used in this study were antibodies to PTEN, Akt, pAkt (ser375) (CST, USA), and the antibody against *β*-actin (Santa Cruz, USA).

### 2.7. Quantitative Real-Time PCR Analysis for miRNA Expression

Using TRIzol (Invitrogen), total RNA was extracted and treated with RNase-free DNase (Qiagen). The researchers conducted mature miRNA expression analysis using a TaqMan Micro-RNA Assay (Applied Biosystems). Next, quantitative reverse transcriptase polymerase chain reaction (qRT-PCR) used the Roche LightCycler480 Real-Time PCR System, with human U6 as an endogenous control. All reactions were done in a 25 *μ*L reaction volume in triplicate. Primers for mature miR-221 miRNA and U6 snRNA were obtained from Ambion. The level of each miRNA expression was measured using the 2^−ΔΔCt^ equation [9]. The expression levels of miR-221 in A549 and H1299 cells were presented as fold changes relative to that in H226. The expression of miR-221 in A549/CDDP cells, which were treated with 50 *μ*M Cisplatin for 72 hours, was presented as fold change relative to that in the parental A549 cells.

### 2.8. Statistical Analysis

Results were expressed as the mean ± standard deviation (SD), and statistical analyses were performed using two-tailed paired or unpaired Student *t-*test or one-way analysis of variance (ANOVA) in GraphPad Prism 5.0 (GraphPad, La Jolla, CA). A *p* value of <0.05 was considered statistically significant.

## 3. Results

### 3.1. miR-221 Is Overexpressed in CDDP-Resistant A549(A549/CDDP) Lung Cancer Cells

**Figure 1:**
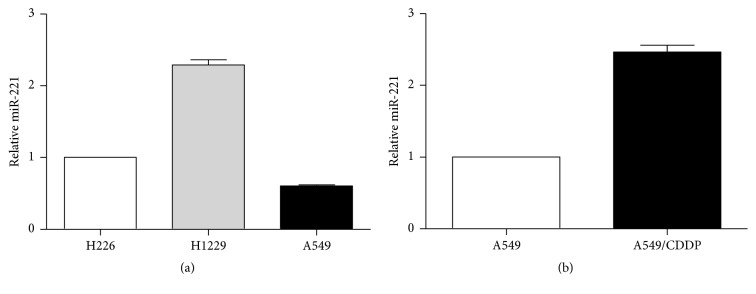
*The expression of miR-221 in A549/CDDP cells*. (a) miR-221 expression in lung cancer cell lines A549, H1299, and H226 by real-time PCR, and U6 was used as an internal control. Results are displayed on fold difference. (b) miR-221 expression was analyzed by real-time PCR in A549 and A549/CDDP cells, and U6 was used as an internal control. Results are displayed on fold difference.

**Figure 2:**
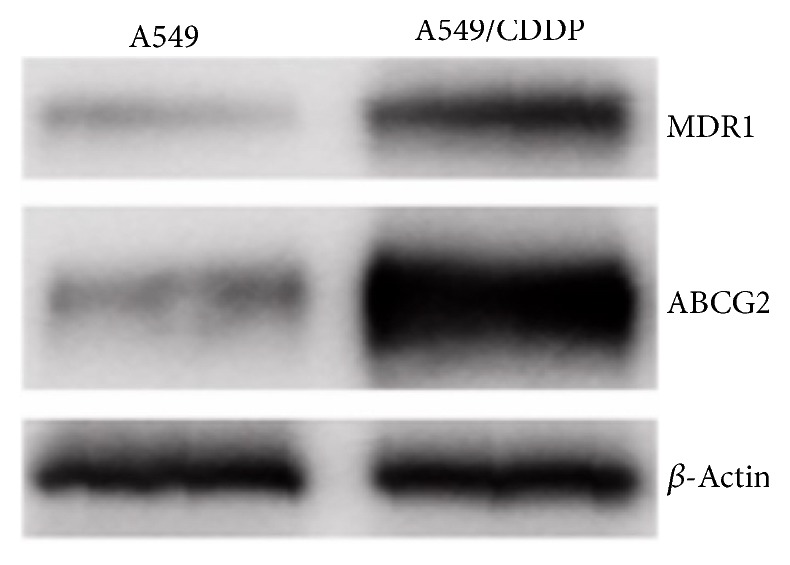
The overexpression of drug-resistant markers. Drug-related proteins MDR1 and ABCG2 were overexpressed in CDDP resistance A549 detected by Western blotting. *β*-Actin was detected as control.

First, we measured the miR-221 expression level in different lung cancer cell lines and found that miR-221 was downregulated in A549 cells and H226, compared to H1299 cells (Figure 1(a)). Compared with parental A549, the expression of miR-221 was higher in A549/CDDP cells (Figure 1(b)). Given that miR-221 showed a higher expression level in CDDP-resistant cancer cells, we explored whether miR-221 may contribute to the CDDP chemoresistance in lung cancer. Our results showed that A549/CDDP was resistant to Cisplatin compared to A549. We also found the overexpression of two drug-resistant markers MDR1 and ABCG2 proteins in CDDP-resistant A549 cells (Figure 2) by Western blot, which verified the chemoresistance properties of CDDP-resistant A549.

### 3.2. miR-221 Renders Lung Cancer Cells Resistant to Cisplatin

Considering the higher expression level of miR-221 in A549/CDDP cells, for this, H1299 cells and A549 cells were transfected with miR-221 mimics or control, respectively. This led us to investigate the effect of miR-221 for chemosensitivity. For this, H1299 cells and A549 cells were transfected with miR-221 mimics or control, respectively. Finally, we discovered that miR-221 was overexpressed (~5-fold) in H1299-miR-221 cells as compared to H1299-Cont cells. miR-221 was inhibited (~3-fold) in A549-anti-miR-221 cells compared to A549-Cont cells.

**Figure 3:**
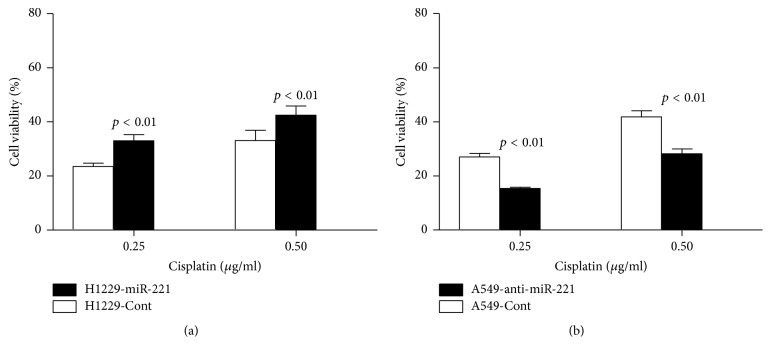
*Inhibition of miR-221 sensitizes lung cancer cells to Cisplatin*. (a) Viability of H1299-miR-221 cells and H1299-Cont cells determined by CCK-8 test. (b) Viability of A549-anti-miR-221 cells determined by CCK-8 and compared to that of A549-Cont cells.

Next, our results showed that the viability of H1299-miR-221 cells was much higher in response to Cisplatin treatments (Figure 3(a)), suggesting miR-221 enhances H1299 cells resistance to Cisplatin. Conversely, the viability of A549-anti-miR-221 cells was much lower following Cisplatin treatment as compared to A549-Cont cells (Figure 3(b)), suggesting that knockdown of miR-221 sensitizes these cells to Cisplatin.

### 3.3. Anti-miR-221 Inhibits Cell Proliferation and Induces Cellular Senescence in Lung Cancer Cells

**Figure 4:**
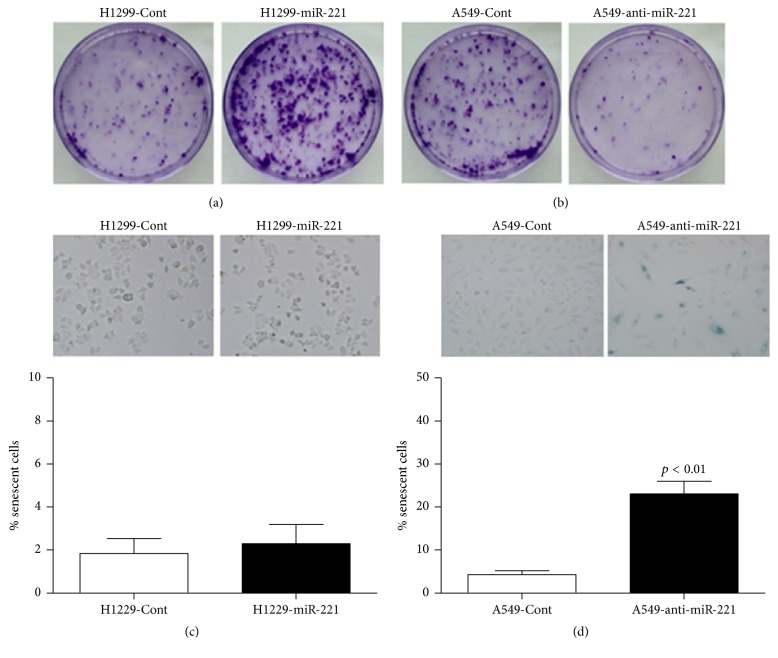
*Cisplatin treatment in miR-221 knockdown A549 cells induces cellular senescence and reduces malignancy.* (a) H1299-miR-221 and H1299-Cont cells were treated with Cisplatin at different points in time, and cell viability was determined by plate colony formation. (b) A549-anti-miR-221 and A549-Cont cells were treated with Cisplatin at different points in time, and cell viability was determined by plate colony formation. (c) H1299-miR-221 and H1299-Cont cells were treated with Cisplatin at different points in time, and cell senescence was detected by SA-*β*-gal assay. (d) A549-anti-miR-221 and A549-Cont cells were treated with Cisplatin at different points in time, and cell senescence was detected by SA-*β*-gal assay. Results are presented as the mean of three separate experiments with standard errors.

Through the plate colony formation assay, we observed that miR-221 induced cell viability (Figure 4(a)) in H1299-miR-221 cells and miR-221 knockdown reduced cell viability (Figure 4(b)) in A549-anti-miR-221 cells, which was consistent with the accepted former results that miR-221 acts as an onco-miRNA. We next investigated the mechanism of cell death increased chemotherapy sensitivity in Cisplatin-treated H1299-miR-221 cells and A549-anti-miR-221 cells. A549-miR-221 cells induced cellular senescence following Cisplatin treatment as compared to A549-Cont cells, detected by SA-*β*-gal assay (Figure 4(d)). By contrast, miR-221 did not have a significant effect on cell senescence (Figure 4(c)). Therefore, it is conceivable that knockdown of miR-221 in A549 cells makes them chemotherapeutically drug sensitive and induces cellular senescence.

### 3.4. miR-221 Regulates PTEN/Akt Expression

**Figure 5:**
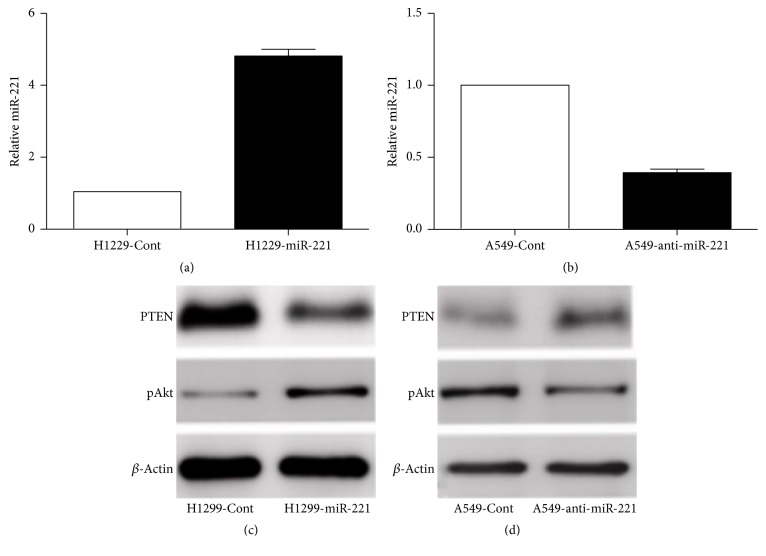
*miR-221 regulates PTEN/Akt pathway*. (a) Expression of miR-221 was examined by PCR in H1299-miR-221 cells and its paired control cells (H1299-Cont). (b) Expression of miR-221 was examined by real-time polymerase chain reaction (PCR) in A549-anti-miR-221 cells and its paired control cells (A549-Cont). (c) Lysates of H1299-miR-221 and its paired control cells (H1299-Cont) were subjected to Western blot analysis using a specific antibody for detection of PTEN and pAkt. (d) Lysates of A549-anti-miR-221 cells and its paired control cells (A549-Cont) were subjected to Western blot analysis using a specific antibody for detection of PTEN and pAkt. Ct values were normalized with control mammalian U6 snRNA (dCt).

To assess the drug resistance mechanism of miR221, we engineered anti-miR221 in A549 cells and found that inhibition of miR-221 sensitizes A549 cells to CDDP (Figure 5(b)). We also inhibited expression of miR-221 in H1299 cells (Figure 5(a)). In order to clarify its mechanism, here we detected the reported miR-221 in the previous studies. And we found that when miR-221 was ectopically expressed, PTEN was decreased and Akt activity was increased in H1299 cells (Figure 5(c)). On the other hand, miR-221 antisense oligonucleotides (ASO) were used to suppress miR-221 activity, and upregulation of PTEN and downregulation of Akt activity in A549 cells were accompanied (Figure 5(d)). As we suspected, suppression of miR-221 could lead to increase of PTEN expression level and enhance the CDDP chemosensitivity.

## 4. Discussion

miRNAs are a group of small noncoding RNAs, which often have about 22 nucleotides. They regulate gene expression by targeting RNAs for translational repression. miRNAs are powerful players in regulating gene expression and the phenotype of tumor cells because of the way they function in cell proliferation and differentiation, in cell cycle progression, and in cell survival, invasion, and morphogenesis [14–18].

In spite of the widely recognized relationship between miRNA and cancer [14–18], the roles and the exact molecular mechanisms of miRNA in the development of cancer drug resistance remain largely unexplored. MCF-7 cells are more resistant to Cisplatin than are other breast cancer cells [19]. In this study, we demonstrated that miR-221 was upregulated in A549/CDDP cells compared to A549 cells, suggesting that miR-221 might play a role in chemotherapy resistance in lung cancer. Furthermore, our research data suggested that knockdown of miR-221 is indeed involved in Cisplatin chemosensitivity in lung cancer cells and even induces cellular senescence. The connection between miR-221 and the establishment of a drug-resistant phenotype in A549/CDDP cells was supported by the correlation between expression of miR-221 and evident changes in the protein levels of their targets, in particular any targets that have been linked to the development of cancer cells drug resistance.

We then observed the enhanced PTEN and reduced pAkt expression in miR-221 knockdown A549 cells. According to our search of the literature, ours is the first report demonstrating the involvement of miR-221 in promoting Cisplatin chemosensitivity. The results of our study suggested that the cellular senescence effect is a key mechanism of miR-221-mediated Cisplatin resistance in lung cancer cells. The contribution that constitutive activation of Akt makes to chemoresistance in different types of tumors has been well confirmed in many reports [20–22]. One of miRNAs family members miR-214 was found by Yang et al. [23] to induce Cisplatin resistance by targeting PTEN. Our study confirmed that miR-221, a new family member of PTEN regulators, blocks PTEN translation leading to activation of the Akt pathway and plays a key role in regulating the Cisplatin chemosensitivity pathway in lung cancer.

However, the induction of cellular senescence effect after overexpression of miR-221 was not observed in H1299 cells, which might be due to the deletion status of p53 in H1299. While p53 is an important inducer of cellular senescence, H1299 might induce cellular senescence by apoptosis. Further research is needed to verify this.

In summary, increased expression of miR-221 in CDDP lung cancer cells and the induction of chemotherapy resistance in lung cancer cells by miR-221 have been demonstrated. Moreover, PTEN was indicated to be the target of miR-221 and is therefore a powerful contributing factor in Cisplatin sensitivity of lung cancer cells. These findings have important implications in the development of targeted therapeutics against Cisplatin resistance in lung cancer.
